# Nonsynonymous Single-Nucleotide Variations on Some Posttranslational Modifications of Human Proteins and the Association with Diseases

**DOI:** 10.1155/2015/124630

**Published:** 2015-10-01

**Authors:** Bo Sun, Menghuan Zhang, Peng Cui, Hong Li, Jia Jia, Yixue Li, Lu Xie

**Affiliations:** ^1^School of Life Science and Biotechnology, Shanghai Jiao Tong University, 800 Dong Chuan Road, Shanghai 200240, China; ^2^Shanghai Center for Bioinformation Technology, Shanghai Academy of Science and Technology, 1278 Ke Yuan Road, Shanghai 201203, China

## Abstract

Protein posttranslational modifications (PTMs) play key roles in a variety of protein activities and cellular processes. Different PTMs show distinct impacts on protein functions, and normal protein activities are consequences of all kinds of PTMs working together. With the development of high throughput technologies such as tandem mass spectrometry (MS/MS) and next generation sequencing, more and more nonsynonymous single-nucleotide variations (nsSNVs) that cause variation of amino acids have been identified, some of which result in the damage of PTMs. The damaged PTMs could be the reason of the development of some human diseases. In this study, we elucidated the proteome wide relationship of eight damaged PTMs to human inherited diseases and cancers. Some human inherited diseases or cancers may be the consequences of the interactions of damaged PTMs, rather than the result of single damaged PTM site.

## 1. Introduction

More than 200 different types of protein posttranslational modifications (PTMs) have been detected. PTMs are involved in many protein activities and cellular processes, such as protein folding, stability, conformation, and some significant regulatory mechanisms [1]. For instance, reversible phosphorylation is involved in conformational changes of enzymes, which results in their activation and deactivation in signaling transduction [2]; the proteins with attached single ubiquitin (Ub) or poly-Ub chains are associated with gene transcription, DNA repair and replication, intracellular trafficking, and virus budding [3]; methylation at certain residues of histones can regulate gene expression [4], and glycosylation is responsible for targeting substrates and changing protein half-life [2].

With the development of high-throughput sequencing technology, gene mutation detection has become another important resource to investigate regulatory mechanisms and cellular processes. Some databases such as dbSNP [5] and SNVDis [6] curated such mutation data. Other secondary databases curated mutation data annotated to the phenotype or diseases, such as Clinvar [7], COSMIC [8], and SwissVar [9]. These databases provide resources to analyze the effect of mutations on human health. However protein activities are closer to disease activities. Either at genomic or at proteomic level, mutations have significant impact on normal gene or protein function, and human diseases could be associated with mutations like nonsynonymous single-nucleotide variations (nsSNVs) on amino acids. Yet how gene mutations affect protein activities through posttranslational modification sites have not been widely studied.

A PTM site that bears nsSNVs can be defined as damaged PTM. Recently, large-scale studies have shown that damaged PTMs caused by numerous inherited and somatic amino acid substitutions [10] have profound impact on both gene and protein function [11], and they are associated with human cancer [12]. One instance is that mutation S215R occurring on the PTMs of TP53 could result in breast cancer [13]; another is mutation of T286 in cyclin D1 (CCND1) causing the loss of phosphorylation of T286 is involved in nuclear accumulation of cyclin D1 in esophageal cancer [14].

However, some of these previous studies concluded the relationship between damaged PTMs and human health based on predications; some focused only on cancers and many focused on only unique type of PTM. Although data of both gene mutations and PTMs are increasing fast, the proteome-wide analysis on the relationship between damaged PTMs and human diseases is not well studied. In this work, we chose eight experimentally demonstrated damaged PTMs to elucidate their association to human diseases including inherited diseases and cancers (somatic diseases). These eight types of damaged PTMs include amino acid variations on Phosphorylation, Ubiquitylation, Acetylation, Glycosylation, Methylation, SUMOylation, Hydroxylation, and Sulfation, which have been well proved to play key roles in important cellular processes and have close relationship with human disease development; moreover, some cross talks among them have been recently revealed in the view of systematic biology [15, 16]. In this study, we focused on the effect of nsSNVs affecting the functions of these eight important normal PTMs and established a new protocol to analyze and view how these damaged PTMs are associated with human diseases.

## 2. Materials and Methods

### 2.1. Datasets

The eight human PTM data sets of Phosphorylation, Ubiquitylation, Acetylation, Glycosylation, Methylation, SUMOylation, Hydroxylation, and Sulfation were obtained from SysPTM 2.0 (released in June, 2013) [17], which integrated PTMs from public resources as well as manually curated MS/MS identified PTMs from experimental research articles, and dbPTM 3.0 (released in June, 2012) [18]. In this study, we only collected human-related PTMs, and we chose the most frequently modified residues for each type of PTM, respectively. For Phosphorylation, we chose His, Ser, Thr, and Tyr; for Ubiquitylation, we chose Lys; for Acetylation, we chose Ala, Gly, Lys, Met, Ser, and Thr; for Glycosylation, we chose Lys, Ser, and Thr; for Hydroxylation, we chose Asn, Pro, and Lys; for Methylation, we chose Lys, Arg; for Sulfation, we chose Ser, Thr, and Tyr; for SUMOylation, we chose Lys.

The inherited-diseases-related nsSNVs were obtained from ClinVar (accessed in November, 2013) [7], dbSNP (build 141) [5], and SwissVar [9]. Cancer-related nonsynonymous single-nucleotide variations (nsSNVs) data were retrieved from COSMIC [8], TCGA (https://tcga-data.nci.nih.gov/tcga/), and SNVDis [6]; neutral nsSNVs were extracted based on dbSNP (build 141) [5], excluding cancer-related SNVs that overlapped with those in COSMIC and TCGA, and other deleterious nsSNVs were filtered by UniProtKB/Swiss-Prot (UniProt released in October, 2013) [19] and PolyPhen-2 [20] which curated credible nsSNVs mapped on UniProtKB. Then we mapped all these nsSNVs to UniprotKB according to the accession number.

### 2.2. Mapping PTM Sites with nsSNV Sites

For phosphorylation mapping, we set three criteria: exact match; ±2 sites around the phosphorylated amino acid; ±7 sites around the phosphorylated amino acid [21]. As for the remaining seven types of PTMs studied, we set two criteria: exact match; ±2 sites around the modified amino acid. For phosphorylation, which is the most widespread type of PTM used in cellular signal transduction [22], in general, protein kinases show a strong selectivity for the primary sequence around the phosphorylation residues such as serine (S), threonine (T), and tyrosine (Y) [12], so we chose the maximum range up to ±7 sites around the phosphorylation sites. However, for ubiquitylation, which is commonly known as a type of PTM that targets proteins for degradation [23], by contrast, little primary sequence selectivity for most E3 ubiquitin ligases surrounding the target Lys was exhibited [15]. For the remaining types of PTMs, such as glycosylation, which is important in protein folding and stability [24] and acetylation, which influences gene regulation in eukaryotic cells [25], in order to unify the range and the numbers of nsSNVs around the modification sites, we all chose the same criteria with ubiquitylation.

### 2.3. Association between Damaged PTM Sites and Diseases

nsSNV affected PTM sites are defined as damaged PTMs in this work. Annotations of nsSNVs (deleterious or neutral) were based on the information from the databases mentioned above and on Online Mendelian Inheritance in Man (OMIM; http://www.ncbi.nlm.nih.gov/omim) [26] for reference. Moreover, we identified the elaborate annotated information of nsSNV-related diseases from SwissVar [9] and the explicit matching of nsSNVs with PTM sites was performed. We calculated the association between damaged PTMs and human diseases based on proteins carrying damaged PTM (with SNV related disease annotation-inherited diseases (germline diseases) or cancers (somatic disease)) for each type of PTM, respectively, by hypergeometric test. In our hypergeometric test, the diseases-associated nsSNVs mapped on or around PTM sites were taken as the test dataset, the neutral nsSNVs mapped on or around PTM sites mentioned above were used as control dataset, and the total neutral nsSNVs and the total damaging nsSNVs on proteins containing one specific type of PTM were used as the two background datasets, to find the disease-associated damaged PTM proteins (with damaging SNVs on this type of PTM) (with *P* < 0.05).

### 2.4. Functional Analysis of Diseases Associated Damaged PTM Sites

To further analyze the functions and features of diseases-related damaged PTMs and their proteins, enrichment analyses were performed using DAVID 6.7 (the database for annotation, visualization, and integrated discovery) [27]. Pathways, biomarkers, and related drugs were analyzed by software Ingenuity Pathway Analysis (IPA) (Ingenuity Systems, http://www.ingenuity.com/). In order to find the structure information of the damaged PTMs, we performed domain enrichment analysis for both inherited disease and cancer-related damaged PTMs based on the domain information from Pfam (version 27.0, released in June, 2012); only the domains containing damaged PTMs were chosen. The enrichment results were calculated and chosen based on disease-related PTM-containing proteins using Fisher's exact test and adjusted with Benjamini-Hochberg method (corrected *P* value < 0.01).

### 2.5. Cross talks between PTM Types

As for the cross talks between some pairwise types of PTMs, positive and negative cross talks were both considered. Positive cross talk means that one PTM serves as a signal for the addition or removal of a second PTM, or for recognition by a binding protein that carries out a second modification. The negative cross talk could be direct competition for modification of one single residue on a protein, or one modification masks the recognition site of a second PTM [27]. Some positive cross talks can be seen from the pathways or networks they are involved in, based on the physical distance and protein-protein interaction, while negative cross talks can be seen on the same residues where different PTMs compete to occur. Nowadays, more and more information of PTMs have been annotated into protein-protein interaction and associated networks [28], and we mined the cross talks between PTMs based on PTMcode 2 (http://ptmcode.embl.de/) which compiles known and predicated PTM associations [29]. The interaction of the eight damaged PTMs with annotated disease information was illustrated with STRING (http://string-db.org/) [30].

## 3. Results

**Figure 1:**
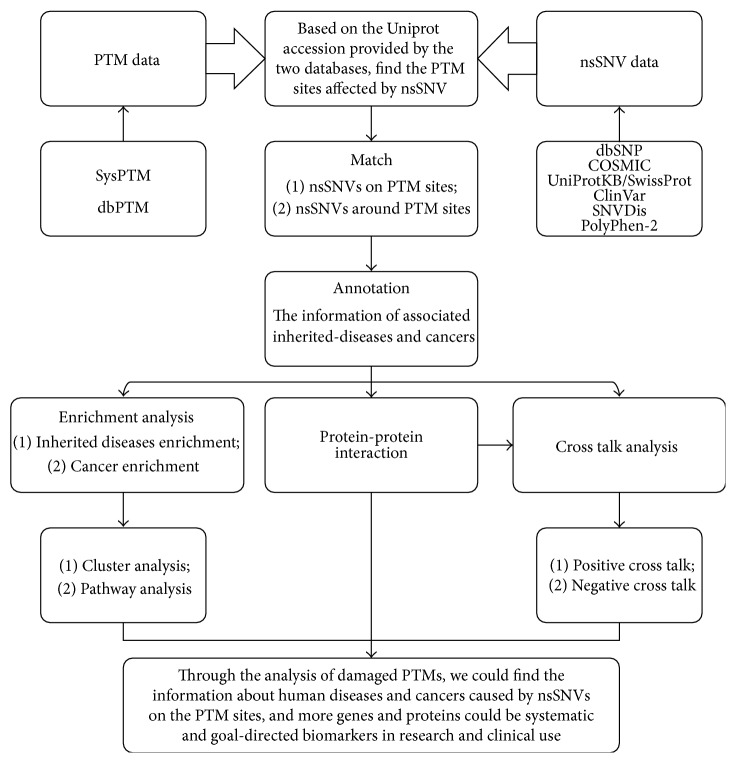
Workflow or protocol for identifying damaged PTMs and associated diseases.

**Figure 2:**
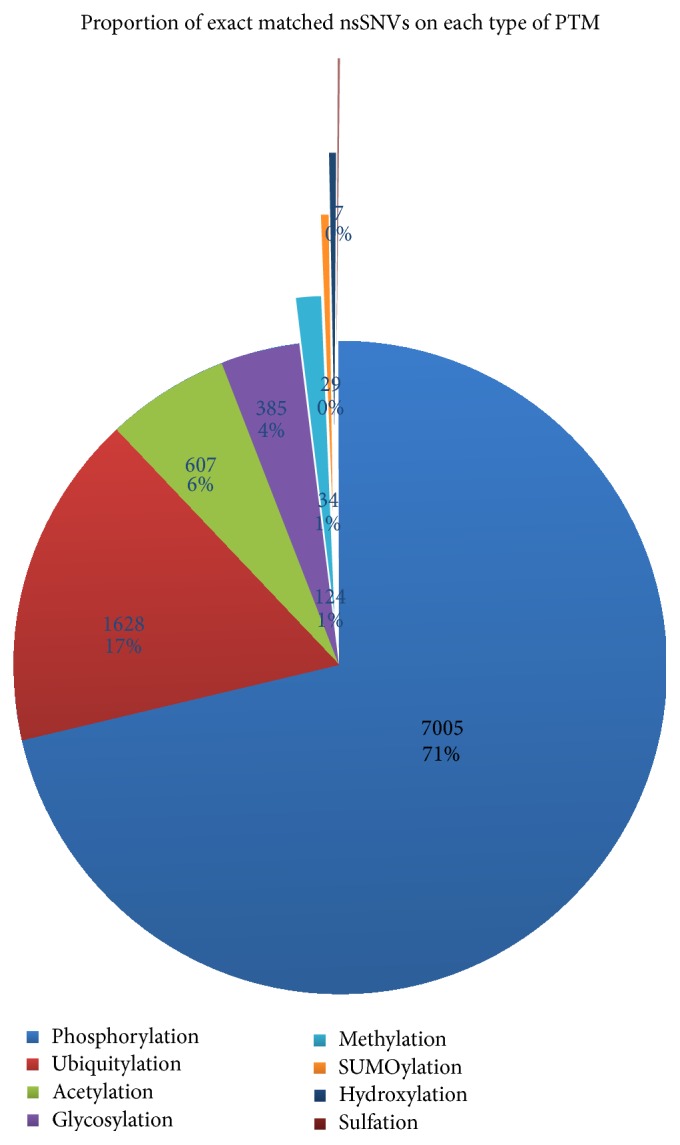
Proportions of exact matched nsSNVs on each PTM out of all sites analyzed. Both the exact number of sites affected and the proportion are shown.

The workflow and protocol of this study are shown in Figure 1. We retrieved PTM data and nsSNVs data from the databases mentioned above. Then we matched them to find the PTM sites affected by nsSNVs (the matched results are available in Table S1 in Supplementary Material available online at http://dx.doi.org/10.1155/2015/124630); the percentages of the exact matched result out of all eight types of PTMs is shown in Figure 2, and the concrete numbers of nsSNVs on each type of PTM are presented in Table 1.

### 3.1. The Statistical Relationship between Damaged PTMs and Inherited Diseases and Cancers

We calculated the PTMs affected by inherited disease and cancer-related nsSNVs, respectively, using hypergeometric test and found that phosphorylation affected by nsSNVs was most significantly related to both inherited diseases and cancers. The next is ubiquitylation; however, based on our calculation, it is not significant in inherited diseases, albeit significant in cancers when performing the exact match. The remaining types of PTMs affected by nsSNVs were not significantly associated with inherited diseases. When we expanded to ±2 amino acids around the modified sites, the damaged PTMs significantly associated with inherited diseases included not only ubiquitylation, but also acetylation and glycosylation. Our results implied that most PTMs affected by nsSNVs were cancer-related, rather than inherited-disease-related (see Tables 1 and 2). This phenomenon might be biased by the data source from big cancer project like The Cancer Genome Atlas (TCGA), Pan-Cancer analysis project [31], and databases like Catalogue of somatic mutations in cancer (COSMIC) [8].

We chose the most frequent modified amino acids, such as Histidine (H), Serine (S), Threonine (T), and Tyrosine (Y) for phosphorylation, Lysine (K) for ubiquitylation, and made a calculation on the frequency of the appearance of nsSNVs on these modified amino acids. We found that the occurring frequency of the modified amino acids affected by nsSNVs was lower compared with their appearance on the whole proteome (data not shown). This demonstrated that the modified amino acids were less affected by mutations. Previous researches showed that PTM sites generally play a key role in normal cellular process like protein-protein interactions and signal transduction and therefore are more stable [15, 32], and our results supported this concept.

Phosphorylation is the best studied and also the most prominent PTM, which has the most abundant data as well [33]. The association between damaged phosphorylation sites and both inherited diseases and cancers is significant, no matter for exact match or for ±2, ±7 amino acids around the phosphorylation sites (Tables 2 and 3). 76736 human phosphorylation sites were obtained in total, out of which only 7005 (9.128%) PTM sites were directly disrupted by nsSNVs. 313 (*P* value = 0.01331) and 2684 (*P* value = 0.01974) out of the 7005 damaged phosphorylation sites were inherited-disease-related and cancer-related, respectively. Therefore, phosphorylation affected by nsSNVs was significantly associated with both inherited diseases and cancers (*P* values < 0.05) (Table 2). For protein kinases, in general, they exhibit a strong selectivity for the primary sequence around the residues they will phosphorylate [33], so ranges of ±2, ±7 residues around the phosphorylated sites were used to find impact by nsSNVs [21] in this study. Ser, Thr, and Tyr can all be phosphorylated; the alterations among these three amino acids can result in diseases, such as S251T in connexin43 (Cx43) protein which is associated with congenital conotruncal anomalies [34] (Table S2, shown in red).

In contrast, ubiquitylation shows little selectivity on primary sequence, such as Lysine, which is highly preferred as the target site of most E3 ubiquitin ligases [15]. So we only chose 2 criteria: exact match and ±2 amino acids around Lysine. Compared to phosphorylation, the ratio of ubiquitylation sites affected by nsSNVs over total ubiquitination sites (7.22%) found on ubiquitylation was lower (22542 ubiquitylation sites, 5988 proteins). There were 1628 exactly matched nsSNVs found on ubiquitylation proteins, only 59 (3.624%, *P* value = 0.08067) were inherited disease-associated and 651 (39.98%, *P* value = 0.01722) were cancer-related sites. For acetylation and glycosylation, both were not found closely related with inherited diseases and cancers (Table 1).

Then, for the remaining four types of PTMs, the numbers of both exact match and ±2 range match were much less than those of the PTMs above, albeit these four types of PTMs are involved in a lot of important cellular processes, and recent works also discovered their related functions and diseases. For instance, SUMOylation proteins are implicated in human diseases including cancers and “Huntington's, Alzheimer's, and Parkinson's diseases”; hydroxylation in Asp110Asn is related with “hemophilia b”; methylation in Arg75Trp is associated with “deafness” [35]; as for sulfation, however, we only identified four mutations in one protein FA8_HUMAN and those were associated with “hemophilia.”

Although we found that a lot of damaged PTMs were related with human inherited diseases and cancers, however, almost half of the data remain to be elucidated on their relationships with human diseases. With more damaged PTMs being annotated and analyzed, their impact over health or disease development may become clearer.

### 3.2. The Damaged PTMs Annotated with Information of Inherited Diseases and Cancers

For all of the eight PTM types studied, we annotated some curated information of diseases based on SwissVar, some annotation information were obtained from the source databases. Although the disease information is up-to-date, the limitation of different databases makes it hard to acquire all the information of known diseases. For instance, inherited-disease-related phosphorylation, “congenital, hereditary, and neonatal diseases and abnormalities,” is the most associated disease based on the analysis of SwissVar on exact matched inherited-diseases-related nsSNVs. The next is “skin and connective tissue diseases” and “nervous system diseases.” However, “neoplasms” account for the most part of the known diseases in ubiquitylation and acetylation.

**Figure 3:**
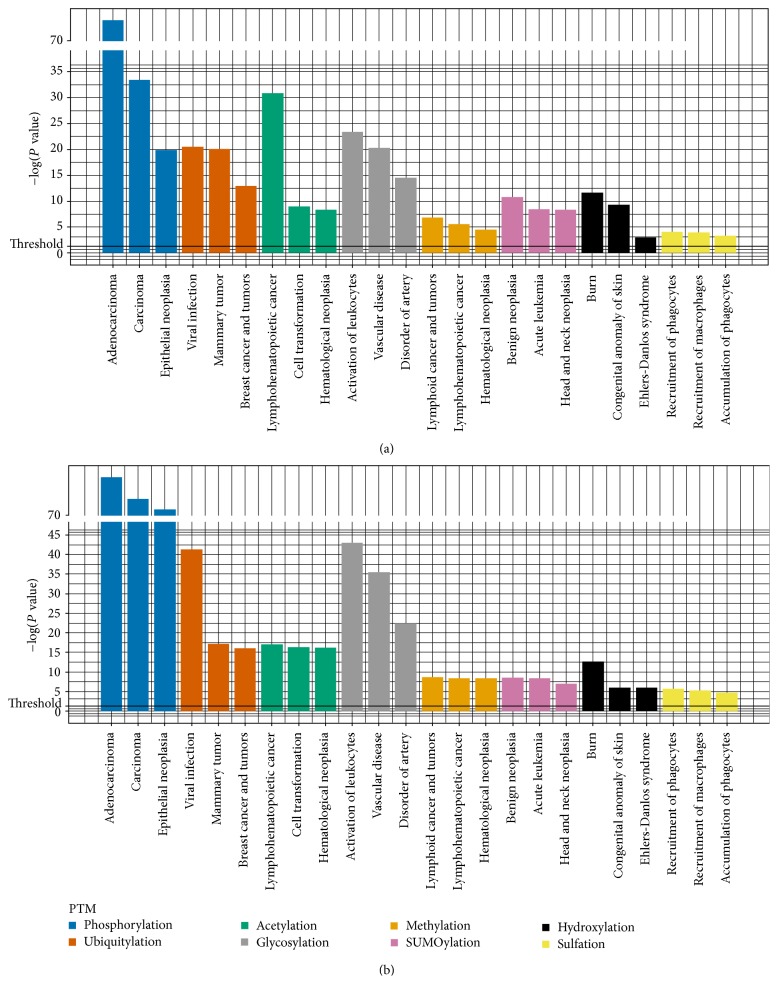
Diseases for each type of damaged PTM affected by nsSNVs in IPA. Threshold was chosen as *P* < 0.05 for all the PTMs and data presented in the charts against −log of *P* values. Different PTMs are shown in different colors. Both (a) and (b) present nsSNVs on the range of ±2 amino acids around modified residues. (a) Diseases for each PTM affected by inherited-disease-related nsSNVs; (b) diseases for each PTM affected by cancer-related nsSNVs.

In order to acquire more information on related diseases, we performed enrichment analysis of diseases using IPA (Figures 3(a) and 3(b)). We performed both inherited-diseases and cancers enrichment analysis on web tool IPA based on the proteins that carried the damaged PTMs, which were caused by the nsSNVs on or around the modification sites. Through enrichment analysis, we could see that in the exact matched phosphorylation related inherited diseases, “autosomal dominant disease” (*n* = 50, corrected *P* value = 5.23*E* − 30,) ranked the first with 50 proteins. For example, PSN1_HUMAN, TNR1A_HUMAN, VHL_HUMA, and PSN1_HUMAN were well studied and associated with “autosomal dominant early-onset Alzheimer's disease” in human [36]. The most significant cancer for the exact matched phosphorylation is “Adenocarcinoma” (*n* = 1074, corrected *P* value = 4.36*E* − 45), which ranked the top with 1074 proteins; RASK_HUMAN, P53_HUMAN, EGFR_HUMAN, and so forth were the representative ones. RASK_HUMAN is associated with adenocarcinoma in human large intestine and lung and other tissues. P53_HUMAN is well known for its associations with human colon and rectal and other cancers [37, 38]; for instance, mutation on Ser376 results in the loss of phosphorylation sites, which creates a consensus binding site for 14-3-3 proteins and increases the affinity of p53 for sequence-specific binding sites on DNA [39]. As to ubiquitylation, “Skin abnormality” was the most significant inherited disease (*n* = 11, corrected *P* value = 3.36*E* − 10), and two proteins were closely related to it: TSC2_HUMAN and TSC1_HUMAN. They were reported to be associated with tuberous sclerosis syndrome in human [40]. Non-small-cell lung cancer was found significant (*n* = 54, corrected *P* value = 4.28*E* − 6) in Ubiquitylation. For acetylation and glycosylation, we also examined both associated inherited diseases and cancers. As to acetylation, we observed disorders of cellular development and cellular growth and proliferation besides cancers that were led by mutations on P53_HUMAN. With regard to glycosylation, the diseases were closely related to lipid metabolism and molecular transport.

We then expanded our search range to the nsSNVs that could affect the PTMs: ±2, ±7 around phosphorylation sites and ±2 for the remaining types of PTMs. First, we chose ±2 range for all the 8 types of PTMs to analyze the associated diseases. For inherited diseases, “autosomal dominant disease” and “autosomal recessive disease” ranked top three in phosphorylation, Ubiquitylation, Acetylation, Glycosylation, Methylation, Hydroxylation, and Sulfation. This was clearly different from the exact matched results. Both autosomal diseases and X-linked hereditary diseases became significant when more nsSNVs were accumulated around PTM sites. The comparison between exact-matched and ±2 range-matched results indicates that (a) mutations on PTMs are rare and, only some certain kinds of inherited diseases were indicated to be caused by them, while more kinds of diseases were indicated to be caused by nsSNVs surrounding PTM sites; (b) human inherited diseases are closely associated with disturbances on and surrounding PTM sites.

Next, we analyzed the ±2 sites range-matched on cancers; the results did not introduce as many changes as exact-matched results. We also compared the data between ±2 and ±7 range around phosphorylation sites; however, their difference was not significant. The differences of human inherited diseases and cancers could be related with the damages of nsSNVs on PTM sites and phenotype: cancers are mostly caused by somatic mutations and present in the current generation; however, the damages of nsSNVs on PTM sites are not easily inherited to the next generation, so the numbers and types of inherited diseases are less compared with damaged-PTM related cancers.

### 3.3. Functional and Structural Analysis

#### 3.3.1. Enrichment Analysis of Keywords, GO, and Domains

We performed functional enrichment analysis using DAVID. First, we performed keywords and GO association analysis (FDR < 0.01). We still divided data into two parts: exact match and ±2 amino acids (AA) match. “Disease mutation” was the most significant keyword based on the inherited-disease-related nsSNVs that appeared in all the four types of PTMs: Phosphorylation, Ubiquitylation, Acetylation, and Glycosylation. The enrichment analyses showed that the proteins we chose were more likely related to diseases when they encountered mutations. GO enrichment analysis was also performed for the four types of PTMs mentioned above. For each PTM category, the differences of functions among them are obvious (see Table S3). For example, the proteins with phosphorylation mainly involve cell activities like cell death, apoptosis, and signal transduction. Coagulation and wound healing were the GO tags for glycosylation. Through the analyses, we found that the diseases led by the damaged PTMs were closely associated with the role of these proteins played in the regulation of normal cellular processes, which indicated that the damage caused by damaged PTMs was serious.

When we moved to cancer-related nsSNVs on PTMs, the keywords about them had less information about mutations, but rather directing to the function of the proteins. What interested us the most was ubiquitylation; the keywords did not show much about themselves, but other modifications on them. This indicates that ubiquitylation is more likely coexisting with other types of PTMs. Then we examined the GO terms on cancers, besides the functions of the proteins performed, also the chemical characters of them showed up. Like phosphorylation, the most significant GO term about phosphorylation was “protein amino acid phosphorylation” on both exact match and ±2 range match. For the remaining types of PTMs, GO terms more revealed protein roles on different processes; for example, “modification-dependent protein catabolic process” ranked in the top two on both range criteria of ubiquitylation.

Then we examined the damaged PTMs associated domains based on the data from Pfam to analyze the impact of damaged PTMs on protein structures. For damaged phosphorylation, “protein tyrosine kinase” (*n* = 13, corrected *P* value = 2.66*E* − 8) and “protein kinase domain” (*n* = 81, corrected *P* value = 2.03*E* − 14) ranked the first in human inherited diseases and cancers, respectively. The damaged phosphorylation on the kinases could result in damage to another phosphorylation and thus nsSNVs do not affect only one phosphorylation site. Then, in terms of ubiquitylation, “P53 DNA-binding domain” (*n* = 8, corrected *P* value = 5.24*E* − 11) and “Histone” (*n* = 11, corrected *P* value = 5.73*E* − 4) were the most significant domains. On P53_HUMAN, lots of phosphorylation and ubiquitylation sites coexisted and some of them affected the same domains, such as “P53 DNA-binding domain.” “Connexin” (*n* = 6, corrected *P* value = 7.06*E* − 7) and “HMG14 and HMG17” (*n* = 18, corrected *P* value = 6.59*E* − 8) were the domains damaged acetylation was enriched in. Glycosylation was involved in wound healing, cell-adhesion, and cellular proliferation and we found that “immunoglobulin domain” (*n* = 8, corrected *P* value = 0.042) and “class I histocompatibility antigen, domains alpha 1 and 2” (*n* = 25, corrected *P* value = 3.97*E* − 4) were enriched in glycosylation domains. Also for Hydroxylation, “collagen triple helix repeat (20 copies)” (*n* = 6, corrected *P* value = 1.06*E* − 9) was found in cancer-related dataset. For other types of PTMs, the domains were scattered compared with PTMs mentioned above. From the data of associated domains, we found that the damaged PTMs associated domains were closely related to molecular binding and protein-protein interactions, which was a major function of PTMs [15].

#### 3.3.2. Pathway Analysis

In order to investigate the function of damaged PTMs in proteome-wide scale, we performed pathway analysis by IPA (details available in Table S4). In IPA analysis for inherited-disease associated damaged PTMs of the exact matched data, some pathways are significant: “ovarian cancer signaling” in Phosphorylation (corrected *P* value = 2.17*E* − 12, ratio = 0.131), Ubiquitylation (corrected *P* value = 3.21*E* − 5, ratio = 0.046), and Acetylation (corrected *P* value = 2.63*E* − 3, ratio = 0.031); “hereditary breast cancer signaling” in Phosphorylation (corrected *P* value = 4.8*E* − 9, ratio = 0.116), Ubiquitylation (corrected *P* value = 6.94*E* − 7, ratio = 0.062), Acetylation (corrected *P* value = 2.36*E* − 3, ratio = 0.036), and Methylation (corrected *P* value = 7.69*E* − 4, ratio = 0.027); “Role of BRAC1 in DNA damage response” in Phosphorylation (corrected *P* value = 1.34*E* − 9, ratio = 0.18), Ubiquitylation (corrected *P* value = 4.72*E* − 4, ratio = 0.066), Acetylation (corrected *P* value = 4.4*E* − 3, ratio = 0.049), and Methylation (corrected *P* value = 6.43*E* − 3, ratio = 0.033). In these pathways, some are associated with their functions like “Coagulation system” (corrected *P* value = 7.75*E* − 10, ratio = 0.171) in glycosylation. As for cancers, we examined each type of PTM category and found that the pathways were more associated with their functions of the proteins, for instance, “protein kinase A signaling” (corrected *P* value = 2.52*E* − 16, ratio = 0.269) in Phosphorylation, “protein ubiquitylation pathway” (corrected *P* value = 9.03*E* − 11, ratio = 0.134) in Ubiquitylation; we found that more cancer-related damaged PTMs were associated with signaling pathways and this indicated that somatic mutations could affect normal cellular processes more often and may thus result in human cancers.

#### 3.3.3. Protein-Protein Interaction Analysis

On the proteome-wide range, the associations among these proteins were close, and we illustrated the interactions using networks of protein-protein interactions with STRING (Figure 5). With a total of 159 proteins which carried identified damaged PTM sites with SwissVar annotated information, we manually divided the associated proteins of different types of PTMs into six major parts, while Sulfation and SUMOylation were not shown for the limited number of data. Not only did some proteins carry one kind of PTMs, such as KRAS, MRE11A, but also phosphorylation, ubiquitylation, and acetylation coexisted on these proteins. From this network, we found that, except for phosphorylation, the interactions among one kind of PTMs were less compared with their interactions with phosphorylation. This result showed us that phosphorylation which was the hub of signal transduction with a strong relationship with other types of PTMs played a key role in the association between damaged PTMs and human inherited diseases and cancers. For example, PTPN11, which was found carrying damaged acetylation caused by (T2I) associated with “noonan syndrome 1” [41], was involved in downstream effectors of cytoplasmic protein tyrosine kinases.

#### 3.3.4. Cross talk Analysis

**Figure 4:**
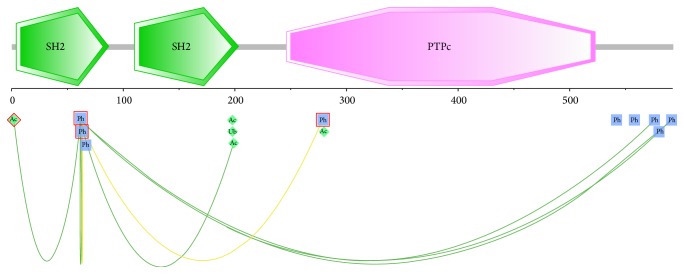
The cross talk of disease-related phosphorylation site Y62 with other PTM sites in protein PTN11_HUMAN. The two “SH2” and one “PTPc” boxed in green and pink are domains in the protein; green lines and yellow lines show the association between PTM sites based on evidence of coevolution and physical distance, respectively. Disease-related PTM sites are boxed in red.

Cross talk between some paired PTMs of different types such as phosphorylation and ubiquitylation and ubiquitylation and acetylation, has become a study theme on proteomics [15, 16]. It shows that the extensive use of PTMs to generate multiple distinct protein states from a single gene product could compensate for the relative paucity of genes in vertebrate genomes [15]. In this work, we investigated the impact of nsSNVs on cross talks between some pairwise PTMs. Cross talks of PTMs can be defined as positive and negative; both mean one PTM has an impact on the other PTM [15]. In this study, we mined the information of cross talks based on PTMcode [29]. Most of the PTM sites have cross talks with other PTM sites based on some evidences such as coevolution and physical distance. Here, we took PTN11_HUMAN as an example for the cross talk within one protein, which totally carried 23 PTMs with 55 functional associations. In our inherited-disease-related dataset, 4 nsSNVs occurred on phosphorylation sites (T2I, Y62D, Y63C, ad Y279C) and 1 on acetylation site of PTN11_HUMAN(Y279S) (Figure 4). The mutations on Y279 are associated with “human LEOPARD syndrome 1” [42], and the mutations on the remaining sites are associated with “human Noonan syndrome 1” [41, 43]; also, within this protein, T2 is associated with both Y62 and Y63, which are all found changed in “Noonan syndrome 1” [41]. Thus, the association of the damaged PTMs could play a key role in the development of human inherited diseases.

**Figure 5:**
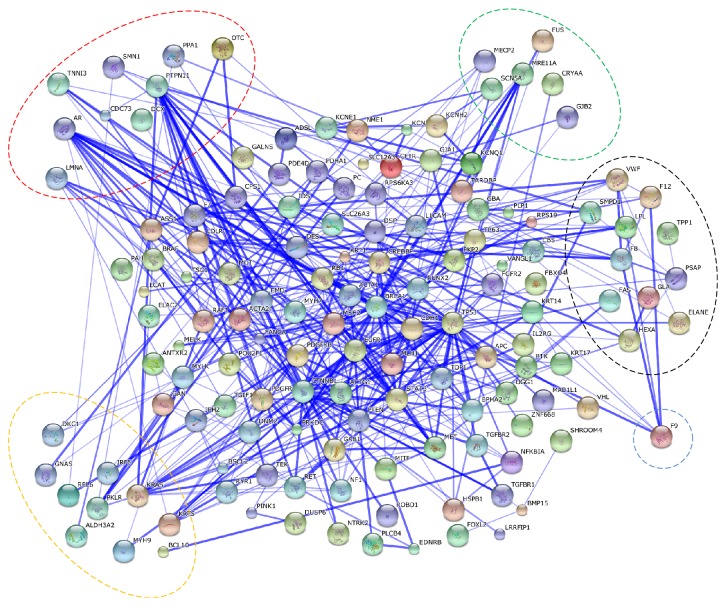
Network of protein-protein interactions among the proteins carrying inherited-disease or cancers related damaged PTMs identified by SwissVar. The proteins were divided into six parts; each category was circled by different colors except for phosphorylation in the center: red represented acetylation, green represented methylation, black represented glycosylation, blue represented hydroxylation and yellow represented ubiquitylation. Stronger associations were represented by thicker lines.

**Figure 6:**
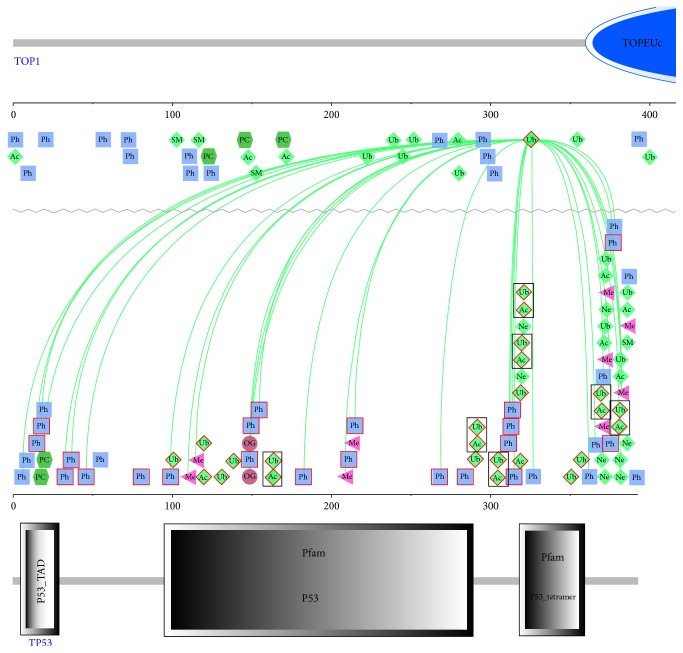
The cross talks between the ubiquitylation site K326 of protein TOP1 with other PTM sites on TP53. Green lines show the association of K326 with other PTM sites based on the evidence of coevolution. Some domains on the two proteins are also given, largely boxed in blue and grey. The different PTMs boxed in red show disease-related PTM sites and those with more than one kind of PTM on the same residue were boxed in black.

On the proteome-wide range, the associations were more prevalent. Then we took P53_HUMAN and TOP1_HUMAN as examples for the cross talks between different PTM sites on distinct proteins: on P53_HUMAN, we found 21 phosphorylation sites, 14 ubiquitylation sites, and 9 acetylation sites; among them, the associations were prevalent within the protein, and the damaged PTMs mostly resulted in the deficiency in the role it played in significant cellular functions [44]; K326R on TOP1_HUMAN is related to human breast cancer [45], and the protein-protein interaction between them is among 159 proteins (Figure 5, boxed in brown); we found that the ubiquitylation on K326 was associated with 33 PTMs in protein P53 (Figure 6); 18 phosphorylation sites were among our inherited disease-related dataset. From the cross talks among these PTMs, we could infer that not only the nsSNVs on one PTM site affect that site, but also other associated sites could be affected. For instance, O-GlcNacylation of S149 in p53 reduces phosphorylation of T155 [15]. Not only human inherited diseases, but also cancers are related to these damaged PTMs.

For the negative cross talk, where more than one kind of PTMs could happen on the same residue, could be occurred in different stage of cellular processes or on different positions. We chose three pairwise PTMs to perform the analysis: phosphorylation and ubiquitylation, phosphorylation and acetylation, and ubiquitylation and acetylation. For the first and second group, phosphorylation and ubiquitylation, and phosphorylation and acetylation, the exact match sites were not overlapped, but when we used damaged ubiquitylation and acetylation sites to match with ±7 sites around phosphorylational sites, we obtained 12 overlapping sites and 10 overlapping sites, respectively, for ubiquitylation and acetylation, and, among them, 7 and 5 sites were on P53_HUMAN, respectively. For example, K320 on TP53 could be ubiquitylated or acetylated (Figure 6). Then we examined the group concerning ubiquitylation and acetylation; we matched their exact sites and obtained 13 overlapping sites. For example, both ubiquitylation and acetylation were detected on K97; nsSNVs on this site could result in “cardiomyopathy, dilated 1a” [46]. Positive cross talk, in which one PTM promotes or prevents another PTM directly on the same site or indirectly on other sites, extends the impact of nsSNVs on PTMs, thus increasing the chance of development of human inherited diseases and cancers in wider ranges. Negative crosstalk with distinct PTMs competing the same site could render nsSNVs on these sites damages to the normal function of all these PTMs, to result in the damages to the related protein functions.

### 3.4. Potential of Damaged PTMs as Biomarkers in Inherited Diseases and Cancers

The damaged PTMs may cause protein functions to be out of control in canonical pathways [47]. For research and medical use, some of them might be very good biomarker candidates [48], which could be used as the drug targets for intervention. We found some proteins with damaged PTMs among the canonical pathways that could be most likely regarded as biomarker candidates using information from IPA. For the exact matched phosphorylation sites with nsSNVs, we filtered 481 gene/proteins; several of them had already been used as the targets of some drugs, but plenty of them still remained to be explored as targets of new drugs (more details available in Table S5). We further identified 169 filtered proteins for ubiquitylation and 90 filtered proteins for acetylation (Table S5). Proteins carrying damaged PTMs are usually associated with lots of critical signaling pathways during the development of diseases [49], such as VHL, which were von Hippel-Lindau tumor suppressor, E3 ubiquitin protein ligase, which was involved in cardiovascular disease, hematological disease, and other diseases. Some of the candidate biomarkers are functionally similar to the known proteins in clinical use. MRP1_HUMAN, which belonged to the family of ABCC1, has been recognized as a biomarker in breast cancer and other cellular disorders [49], with drugs like “sulfinpyrazone.” For each PTM, we provided some most likely biomarkers as candidates (Table S5).

## 4. Conclusions

In summary, through this work, we investigated the associations between PTMs affected by nsSNVs and human inherited diseases and cancers from diverse perspectives such as functions, pathways, and cross talks. These provided us a proteome-wide view of how the proteins, which carry modifications and nsSNVs, play roles in the development of diseases and cancers. Not only do PTMs play key roles in almost every important cellular process, but also their dysfunction could result in human diseases. We provided a practical protocol to analyze disease-related proteins that carry damaged PTMs; some valuable proteins were listed out as the candidate biomarkers for potential research and clinical use. However, still almost half of damaged PTMs did not demonstrate associations with human health based on our current analysis, and their functions need to be revealed. Moreover, what we need to do in the future is to identify the causative relationships between the damaged PTMs and human diseases, by discovering key nsSNVs on protein modifications.

## Supplementary Material

Supplementary Table S1: The proteins and their sites of exact matched nsSNVs on each type of PTM. The proteins were shown with their UniProt Accession number.
Supplementary Table S2: The information of damaged PTM sites associated diseases annotated by SwissVar. For each type of PTM, the information of associated diseases for the exact matched and around matched PTM sites were given. The alterations between the modified amino acids of one type of PTM marked in red.
Supplementary Table S3: Enrichment results of keywords and GO. Both exact matched and ±2 matched results were shown. Inherited diseases were marked in yellow and cancers were marked in green.
Supplementary Table S4: Pathway analysis based on IPA. The results were boxed for each type of PTM, and the analysis were performed for both inherited diseases and cancers. *P* value and the ratio calculated based on IPA were shown.
Supplementary Table S5: Summary of biomarker candidates. The biomarker candidates were chosen based on information from IPA and details about them were shown.

## Figures and Tables

**Table 1:** Numbers of nsSNVs on each PTM category.

PTM	# of exact match	# of ±2 match	# of ±7 match
Phosphorylation	7005	33119	78123
Ubiquitylation	1628	10096	—
Acetylation	607	9642	—
Glycosylation	385	2199	—
Methylation	124	427	—
SUMOylation	34	231	—
Hydroxylation	29	328	—
Sulfation	7	26	—

**Table 2:** Numbers and *P* values of exact matched nsSNVs related to inherited diseases and cancers on each PTM type.

PTM	Inherited disease	*P* value^*∗*^	Cancer	*P* value
Phosphorylation	313	0.0133	2684	0.0197
Ubiquitylation	59	0.0807	651	0.0172
Acetylation	34	0.0701	233	0.1058
Glycosylation	13	0.2062	57	0.1813
Methylation	15	0.1638	67	0.0912
SUMOylation	1	0.7752	22	0.0152
Hydroxylation	2	0.7423	14	0.3507
Sulfation	4	0.5503	0	0.5503

^*∗*^ *P* values in this column were calculated using hypergeometric test and all values refer to the left column (genetic disease).

**Table 3:** Numbers and *P* values of ±2 AA matched nsSNVs related to inherited diseases and cancer on each PTM.

PTM	Genetic disease	*P* value^*∗*^	Cancer	*P* value
Phosphorylation	1422	2.51*E* − 04	12826	0.0111
Ubiquitylation	439	5.59*E* − 03	4074	4.9*E* − 04
Acetylation	552	3.93*E* − 07	4019	8.21*E* − 03
Glycosylation	214	0.0261	795	1.26*E* − 37
Methylation	44	0.1036	231	1.57*E* − 02
SUMOylation	11	0.1526	115	7.93*E* − 06
Hydroxylation	22	0.1997	63	2.82*E* − 03
Sulfation	7	0.2446	9	0.2526

^*∗*^ *P* values in this column were calculated using hypergeometric test and all values refer to the left column (genetic disease).

## Abbreviations

PTM: Protein posttranslational modification
nsSNVs: Nonsynonymous single-nucleotide variations
GO: Gene Ontology
TCGA: The Cancer Genome Atlas
CCND1: Cyclin D1
AA: Amino acid.
